# Supplementary material for: Dandelion-Like CuCo2O4@ NiMn LDH Core/Shell Nanoflowers for Excellent Battery-Type Supercapacitor
Source: Nanomaterials (Basel). 2023 Feb 14;13(4):730. doi: 10.3390/nano13040730 (PMC9967973; doi:10.3390/nano13040730)
Supplement: Supplementary file 1 [file nanomaterials-13-00730-s001.zip › nanomaterials-2183201-supplementary.pdf]

Supplementary Materials

Dandelion-Like $CuCo_2O_4$@ NiMn LDH Core/Shell Nanoflowers for Excellent Battery-Type Supercapacitor

Wenhua Zhao [1,2], Xingliang Xu [1,*], Niandu Wu [2], Xiaodie Zhao [1] and Jiangfeng Gong [3,*]

[1] Department of Applied Physics, Zhejiang University of Science and Technology, Hangzhou 310023, China
[2] National Laboratory of Solid-State Microstructures, Collaborative Innovation Center of Advanced Microstructures, School of Physics, Nanjing University, Nanjing 210093, China
[3] College of Science, Hohai University, Nanjing 211199, China
* Correspondence: xlxu@zust.edu.cn (X.X.); jfgong@hhu.edu.cn (J.G.)

2. Materials and Methods

2.3. Electrochemical test

Ni foam (1 × 1 cm) was washed using acetone, 3 M HCl and absolute alcohol to clean NiO on the surface of Ni foam. Pt, HgO electrode (supercapacitor), and Ni foam with sample were used as a counter electrode, reference electrode, and working electrode in a three-electrode system. The mass of carbon black and polytetrafluoroethylene in accordance with a ratio of 8:1:1. 40 μL of N- methyl pyrrolidone (NMP) was evenly added and coated on the cleaned Ni foam. Then, the Ni foam was dried at 10 h at 80 °C, which was pressed with a pressure of 5 MPa and maintained for 5 min. The loading amount of the working electrode is 2 mg cm^{-2}. The supercapacitor test was in a 3M KOH solution at room temperature.

ASC was assembled with CCO-NM LDH NFs as the cathode and AC as the anode, respectively. The electrochemical behavior was evaluated in a 3M KOH solution.

The energy density corresponding to the power density of the assembled asymmetric capacitor device can be obtained from the formulas:

$$E=0.5\times C\times V/3.6, \quad (S1)$$

$$P = 3600\times E/\Delta T \quad (S2)$$

The columbic efficiencies (η) were determined using the following equation.

$$\text{Columbic efficiency } (\eta) = \frac{\text{Discharging time } (d_t)}{\text{Charging time } (c_t)} \times 100 \quad (S3)$$

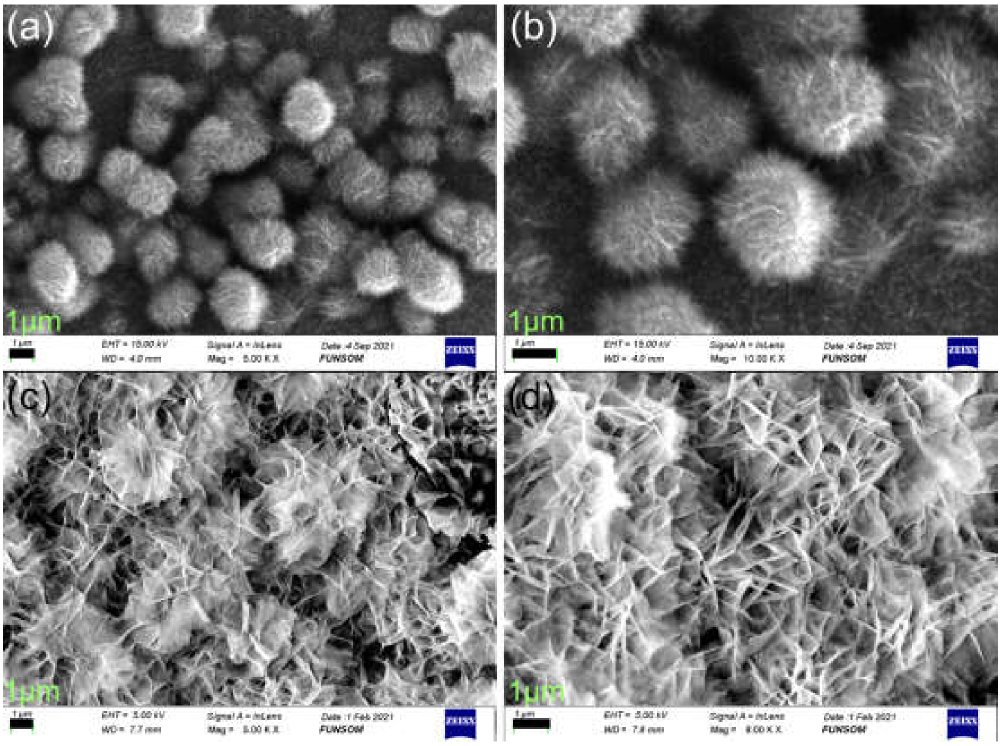

Figure S1. SEM of (a,b) $CuCo_2O_4$ sample and (c,d) $CuCo_2O_4$@NiMn LDH sample.

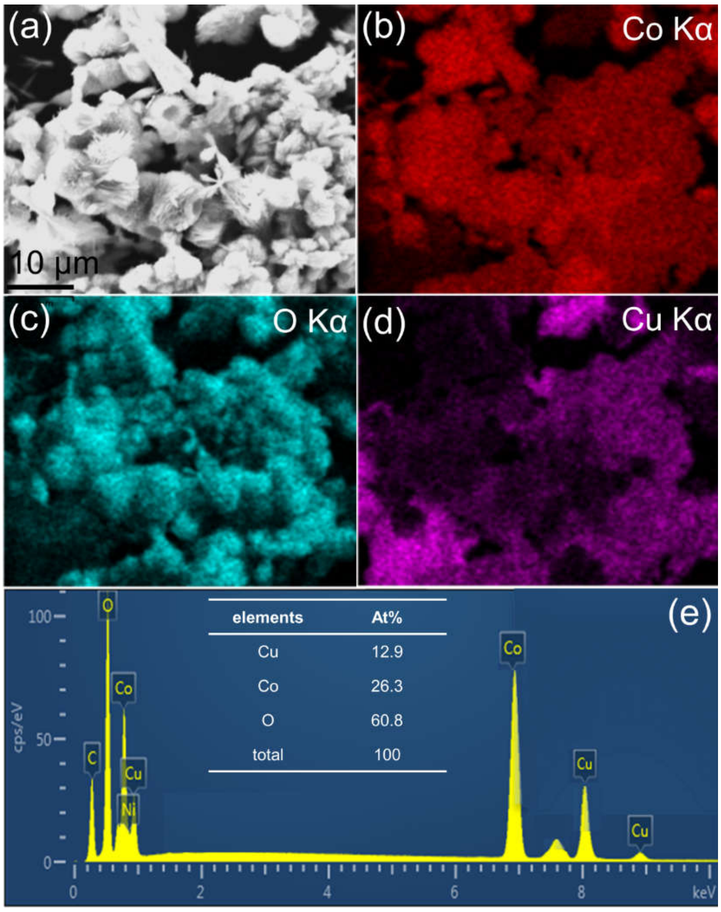

Figure S2. Mapping (a–d) and EDS (e) of $CuCo_2O_4$ sample.

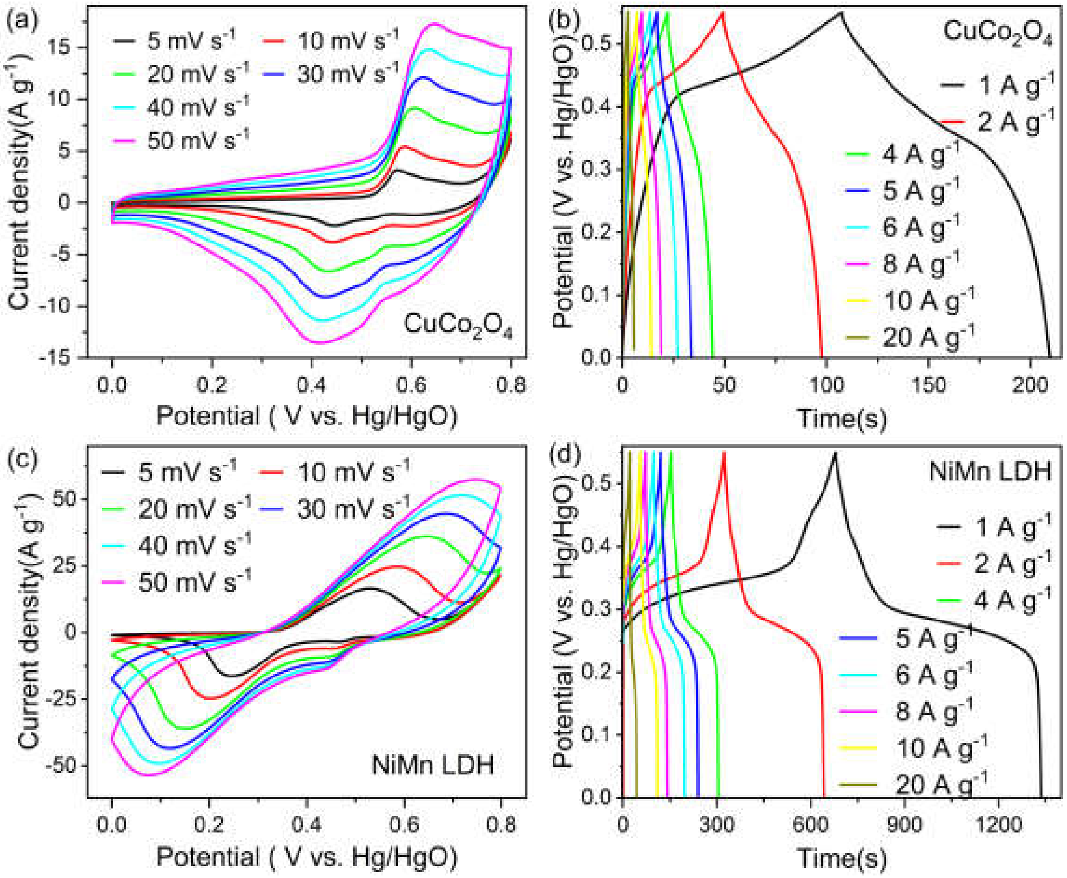

Figure S3. GCD plots at different current densities and CV plots at different scan rates of (a,b) $CuCo_2O_4$ and (c,d) NiMn LDH, respectively.

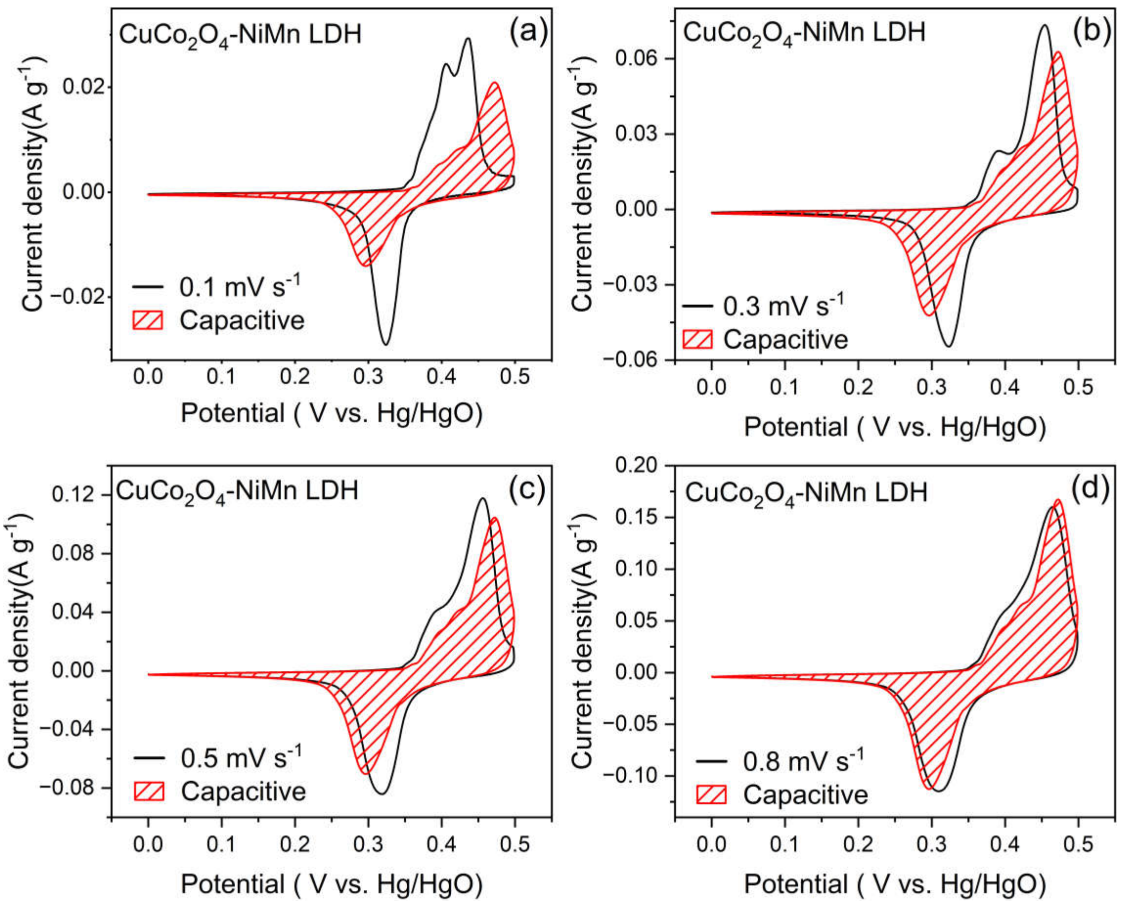

Figure S4. Cyclic Voltammetry profiles were collected at the scan rate between (a–d) 0.1 to 0.8 mV s^{-1} for the $CuCo_2O_4$@NiMn LDH. The capacitive- and diffusion-controlled contribution to the current response is described by pink and white areas.

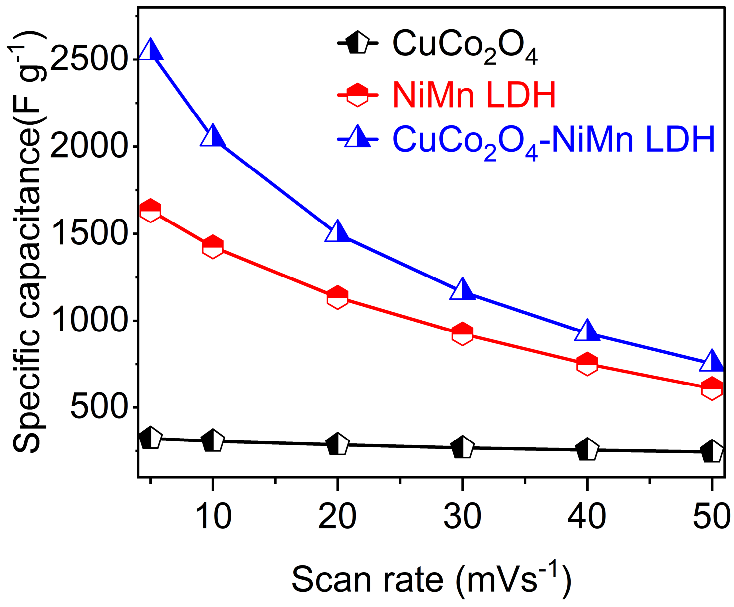

Figure S5. Variation of specific capacitance with scan speed for all samples.

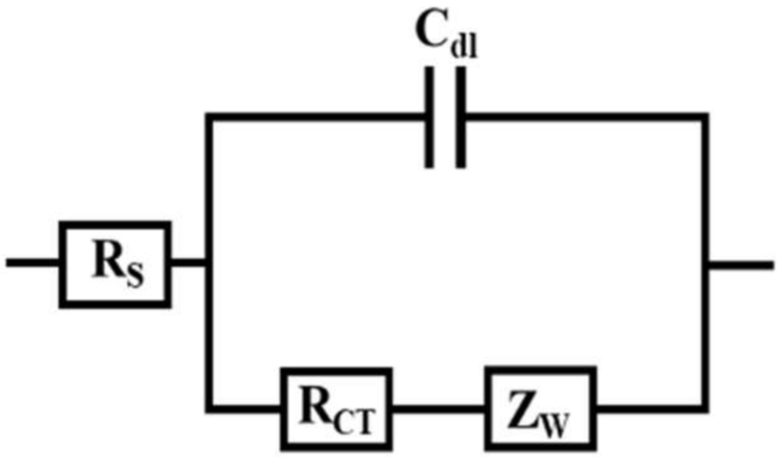

Figure S6. EIS circuit diagram.
